# Efficiency of Disease and Disease Activity Diagnosis Models of Systemic Lupus Erythematosus Based on Protein Array Analysis

**DOI:** 10.1155/2022/1830431

**Published:** 2022-08-04

**Authors:** Yafei Zhao, Yuanyuan Qi, Xinran Liu, Yan Cui, Zhanzheng Zhao

**Affiliations:** ^1^Nephrology Hospital, The First Affiliated Hospital of Zhengzhou University, Henan 450052, China; ^2^Institute of Nephrology, Zhengzhou University, Henan 450052, China

## Abstract

**Background:**

Systemic lupus erythematosus (SLE) has become increasingly common in the clinic and requires complicated evidence of both clinical manifestations and laboratory examinations. Additionally, the assessment and monitoring of lupus disease activity are challenging. We hope to find efficient biomarkers and establish diagnostic models of SLE.

**Materials and Methods:**

We detected and quantified 40 proteins using a quantitative protein array of 76 SLE patients and 21 healthy controls, and differentially expressed proteins were screened out by volcano plot. Logistic regression analysis was used to recognize biomarkers that could be enrolled in the disease diagnosis model and disease activity diagnosis model, and a receiver operating characteristic (ROC) curve was drawn to evaluate the efficiency of the model. A nomogram was depicted for convenient and visualized application of our models in the clinic. Decision curves and clinical impact curves were also plotted to validate our models.

**Results:**

The protein levels of TNF RII, BLC, TNF RI, MIP-1b, eotaxin, MIG, MCSF, IL-8, MCP-1, and IL-10 showed significant differences between patients with SLE and healthy controls. TNF RII and MIP-1b were included in the SLE diagnosis model with logistic regression analysis, and the value of the area under the ROC curve (AUC) was 0.914 (95% confidence interval (CI), 0.859-0.969). TNF RII, BLC, and MIP-1b were enrolled in the disease activity diagnosis model, and the AUC value was 0.823 (95% CI 0.729-0.916). Both of the models that we established showed high efficiency. Additionally, the three protein biomarkers contained in the disease activity distinguish model provided additional benefit to conventional biomarkers in predicting active lupus.

**Conclusions:**

The disease diagnosis model and disease activity diagnosis model that we developed based on protein array chip results showed high efficiency in differentiating patients with SLE from healthy controls and recognizing SLE patients with high disease activity, and they have also been validated. This implied that they might greatly benefit clinical decisions and the treatment of SLE.

## 1. Introduction

Systemic lupus erythematosus (SLE) has always been a complicated systemic autoimmune disease with a high relapse tendency. It is characterized by disorders of the immune system, such as persistently activated T cells and B cells that secrete autoimmune antibodies, dysfunction of macrophages, and abnormally deposited immune complexes [1–4]. It is also known to us because of common and serious complications, for example, lupus nephritis, which could result in hospitalization and death [5]. It has been troublesome and consumes a high economic cost [6]. The diagnosis criterion that we most commonly used in the clinic is 1997 American College of Rheumatology (ACR) revised criteria for the classification of SLE, which included many indices, and is not convenient to use [7, 8]. Evaluation of SLE disease activity has similar issues with that of diagnosis criteria, and it also lacks efficient means of monitoring disease activity and relapse. This urges us to find novel methods for SLE diagnosis and disease activity assessment. Currently, SLE patients show various degrees of abnormal cytokine levels, and some of the secretory protein levels are related to lupus disease activity or systemic organ injuries [9, 10]. Although the discovery of new biomarkers has attracted the interest of researchers, few of the biomarkers that have been found could be used in clinical treatment [11]. However, our attempts and perseverance are still required, and we have confidence in this area. Thus, we used inflammatory proteins as our starting point. We detected and quantified a selected array of proteins and established models for diagnosing SLE and active lupus. Models have also been proven effective, and we believe that our models would be helpful to clinicians and benefit our patients.

## 2. Materials and Methods

### 2.1. Study Population, Subjects, and Ethics Approval

In this clinical study, we recruited seventy-six SLE patients who were hospitalized at the Zhengzhou University First Affiliated Hospital from July 2019 to December 2019. All of the SLE patients we enrolled were diagnosed with SLE based on the 1997 ACR revised criteria for the classification of SLE [7, 8]. The exclusion criteria were (a) suffering an active infection and (b) suffered or suffering a tumor or cancer. At the same time, we recruited a group of 21 healthy controls. To satisfy our study needs, we obtained a 3.0 mL peripheral blood sample from each of our participants in the morning after an eight-hour overnight fast after the day they were enrolled. Blood samples were collected in an EDTA-K2 anticoagulant tube and then centrifuged at 3000 rpm for 10 minutes. The plasma was separated and stored at -80°C until testing. All operations were completed in one hour after the plasma sample was collected. The study was censored and approved by the Medical Ethics Committee of Zhengzhou University First Affiliated Hospital (2019-KY-134). All of the participants enrolled, including all the patients and/or their legal guardians and healthy volunteers, signed written informed consent forms.

### 2.2. Date Collection and Assessment of Inflammatory Protein Levels

The data we collected from SLE patients mainly included demographic information, clinical manifestations, and laboratory and imaging examinations, and all the information above was collected during hospitalization. We assessed and quantified 40 plasma inflammatory protein expression levels in 97 plasma samples (76 SLE patient samples and 21 healthy control samples) by Quantibody® Human Inflammation Array 3 (Cat# *QAH-INF-G3-4*, RayBiotech). All the plasma was thawed for the first time to avoid adverse effects that repeated freeze/thaw cycles might bring to the sample quality. Lupus clinical disease activity was measured based on Systemic Lupus Erythematous Disease Activity Index (SLEDAI) 2000 [12].

### 2.3. Statistical Analysis

Proteins are shown in a scatter diagram and sorted into upregulated, downregulated, and nonsignificant groups based on protein level fold change (FD) of SLE patients compared to healthy controls. Volcano plots and heatmap were also depicted so that we could recognize those differentially expressed inflammatory proteins and intuitively display protein level differences. To identify proteins that could be used to develop a diagnostic model for differentiating SLE patients from healthy controls, we carried out univariable and multivariable logistic regression analyses. We drew a receiver operating characteristic (ROC) curve and calculated the area under the ROC curve (AUC) to evaluate the model efficiency. A logistic regression analysis calibration curve was also performed to further assess diagnostic efficiency and application value. A nomogram is also presented. Decision curves and clinical impact curves were depicted to evaluate and illustrate the clinical application value of our model. Not only the diagnosis model but also a model that would discriminate active lupus patients from inactive ones was established and assessed in similar ways. SLE patients were sorted into active lupus with SLEDAI ≥ 5 and inactive lupus with SLEDAI < 5.

All statistical analyses and figures were generated and drawn using R software version 4.0.5. The variation between groups was considered statistically significant if the two-sided *p* value was less than 0.05.

## 3. Results

### 3.1. Levels of Inflammatory Proteins

We quantified both patients with SLE and healthy controls with a protein array, and all the members came from central China and showed similar sex compositions, which would greatly influence disease activity (shown in Table 1). The median age of controls was less than that of patients with SLE; most of our participants were middle-aged and all were younger than 60 years old. Reports available showed that immune system function would decay until people were older than 60-65 years old [13, 14]. Therefore, we think this heterogeneity would not be a significant factor in our study and conclusion.

**Figure 1:**
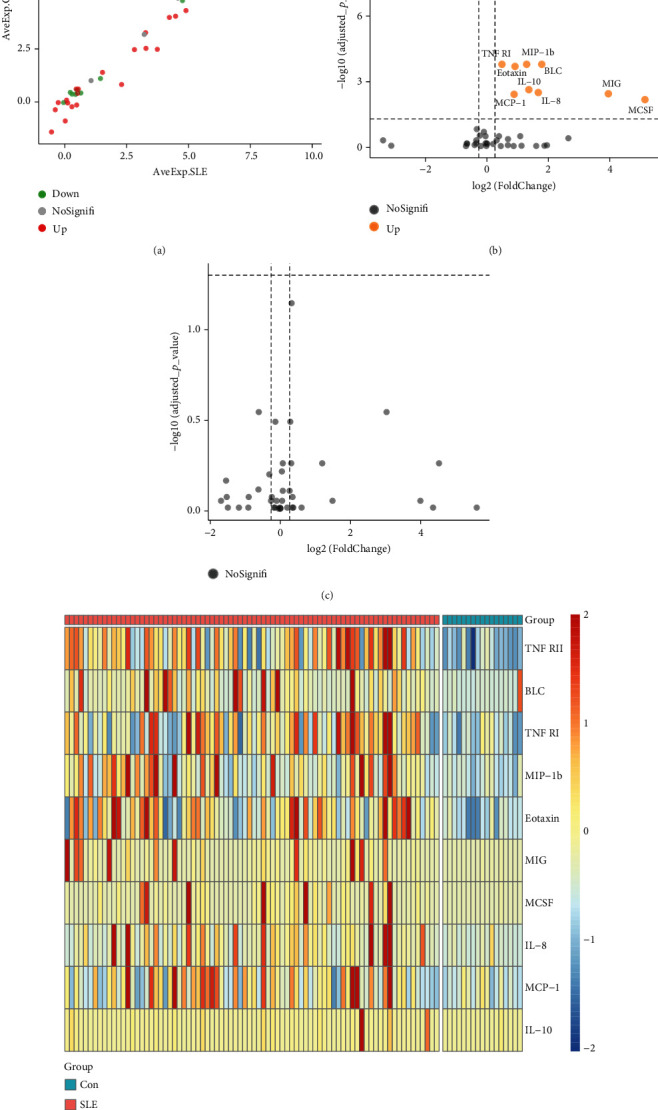
Levels of quantified proteins. (a) Quantified levels of the 40 proteins. Upregulation (red dot) was FD > 1.2, downregulation (green dot) was FD < 0.83, and other proteins (grey dot) were regarded as there was no significant difference between SLE patients and healthy controls. (b) Volcano plot of the 40 proteins and statistical significance between patients with SLE and healthy controls. Adjusted *p* value was determined by method “BH” based on the original p value. (c) Volcano plot of the 40 proteins and differences between SLE patients with and without renal involvement. (d) Heatmap of the ten differently expressed proteins.

To visualize our protein array results, we drew a scatter diagram (Figure 1(a)). AveExp., which represents the average value of logarithmic levels of each protein, determined their position. The average value of SLE patients is shown on the *x*-axis, and that of healthy controls is shown on the *y*-axis. FD was determined by the ratio of the average protein level of SLE patients to healthy controls, and proteins were sorted into different groups based on FD.

We further explored whether these upregulated and downregulated proteins were significantly different between SLE patients and healthy controls, and volcano plot analysis was performed (Figure 1(b)). Log2 (FD) and -log10 (adjusted *p* value) determined the coordinate axis system. According to the plot, we found that TNF RII, BLC, TNF RI, MIP-1b, eotaxin, MIG, MCSF, IL-8, MCP-1, and IL-10 showed significant differences (adjusted *p* value < 0.05), and all of them were upregulated. Detailed information of these ten proteins is shown in Table 2. A heatmap was also displayed to show the expression level differences of these ten proteins (Figure 1(d)), and we found that most high protein levels were obviously distributed in the SLE group.

Additionally, we carried out subgroup analysis to determine whether there were significant protein level differences between SLE patients without renal involvement and lupus nephritis patients [15]. Unfortunately, the results showed all of the adjusted *p* > 0.05, although a few basic *p* values were promising (Figure 1(c)).

Ahead of further analysis, we wanted know whether SLE patients under a given medical regimen would show different levels of the ten selected plasma proteins. We sorted SLE patients into two groups, patients under given regimens (use of glucocorticoids and/or immunosuppressive agents) and patients without a given regimen. Nine of the ten proteins showed similar levels between the two groups, and only MIP-1b in patients under the given regimens was lower than the others (*p* < 0.05). Although the level difference was reduced, patients with SLE still showed higher plasma MIP-1b levels than healthy controls, and this would not change our conclusion.

### 3.2. Development of a Disease Diagnosis Model for Differentiating SLE Patients from Healthy Controls

To identify whether the ten proteins could be used in diagnosing SLE and developing a disease diagnosis model, we carried out univariate logistic regression analysis, and TNF RII, BLC, TNF RI, MIP-1b, eotaxin, MIG, IL-8, and MCP-1 showed statistical significance in distinguishing SLE patients from healthy controls (Table 3). Furthermore, with backward stepwise multiple logistic regression analysis, TNF RII and MIP-1b were finally indicated to be independent risk factors for SLE. Thus, we enrolled TNF RII and MIP-1b in a disease diagnosis model to differentiate SLE patients from healthy controls. The model suggested that people with higher plasma levels of TNF RII and/or MIP-1b tended to suffer SLE.

**Figure 2:**
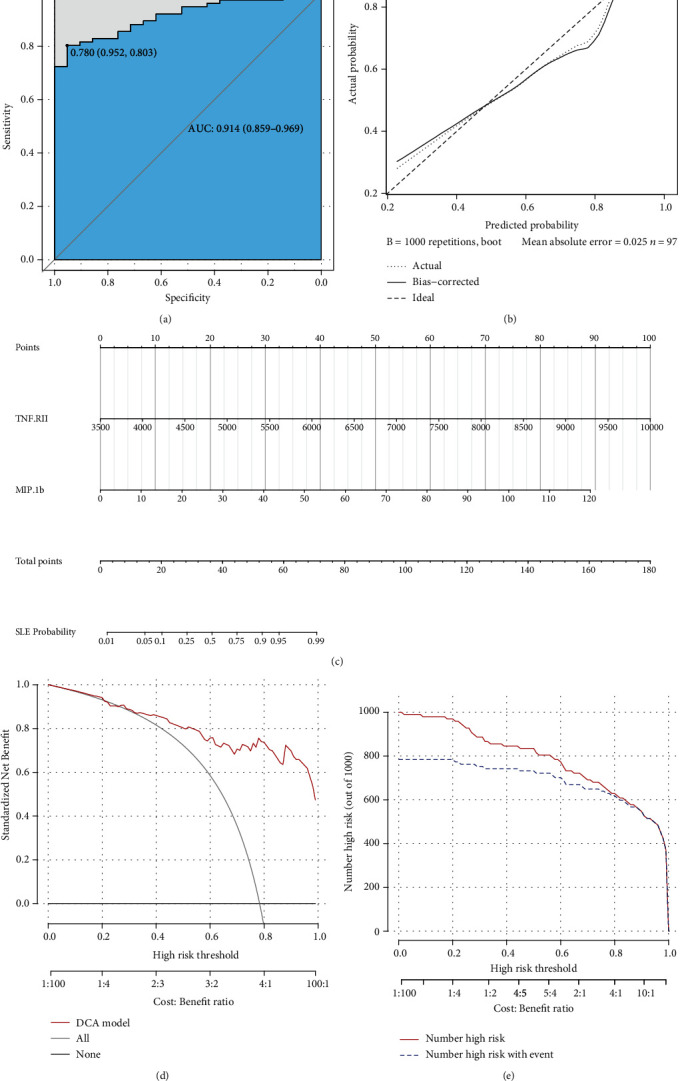
Efficiency and validation of the disease diagnosis model. (a) ROC of diagnosis model in differentiating SLE patients from healthy controls. (b) Calibration curve of the diagnosis model. (c) Nomogram based on the diagnosis model. (d) Decision curve for the diagnosis model in predicting SLE. Standard net benefit (*y*-axis) and risk threshold (*x*-axis) formed the coordinate system. The red line represented our model, grey line represented the assumption that all the people were suffering SLE, and black line represented the assumption that all the people were healthy. (e) Clinical impact curve analysis diagram. Red line represented number of people which were diagnosed with SLE by our model at different threshold probability, and blue line represented number of SLE patients.

Next, we plotted an ROC curve to assess the diagnostic efficiency of the model (Figure 2(a)). It showed a relatively high efficiency with an AUC of 0.914 (95% CI 0.859-0.969), and the cutoff point value was 0.780, with a specificity of 0.952 and a sensitivity of 0.803. Both the high AUC value and specificity and sensitivity of the cutoff point showed a quite high level of discrimination ability. Then, we displayed a calibration curve and manifested a mean absolute error of 0.025, which suggested that this model has a high capability of calibration (Figure 2(b)).

Furthermore, we depicted a nomogram for better use of this model in clinical decision-making (Figure 2(c)). We obtained a score by drawing a vertical line upward from each variable and obtained a point on the “points” axis. Then, we summed the two points and obtained a total point, and we obtained a probability on the “SLE probability” axis. In this way, we could conveniently speculate the probability of suffering SLE based on quantified levels of TNF RII and MIP-1b.

To better evaluate the potential of this model in clinical decision-making, we carried out decision curve analysis (DCA) and drew the curve down (Figure 2(d)). This result suggested that this diagnosis model could add more benefit in the clinic if we could obtain a threshold probability higher than 0.26. Clinical impact curve analysis also showed a high efficiency of our model in distinguishing SLE patients from healthy controls at all risk thresholds (Figure 2(e)).

### 3.3. Combined Protein Markers in Recognizing Active Lupus

How to find a better way to monitor SLE disease activity has always been a hot topic, and we further analyzed the potential of the proteins in predicting active lupus. TNF RII, BLC, and MIP-1b showed statistical significance in logistic regression analysis in differentiating active lupus from inactive lupus (Table 4). This result suggested that SLE patients with high levels of these three proteins were more prone to suffer an active disease, and thus, we established a model for predicting patients with active lupus based on these three proteins.

**Figure 3:**
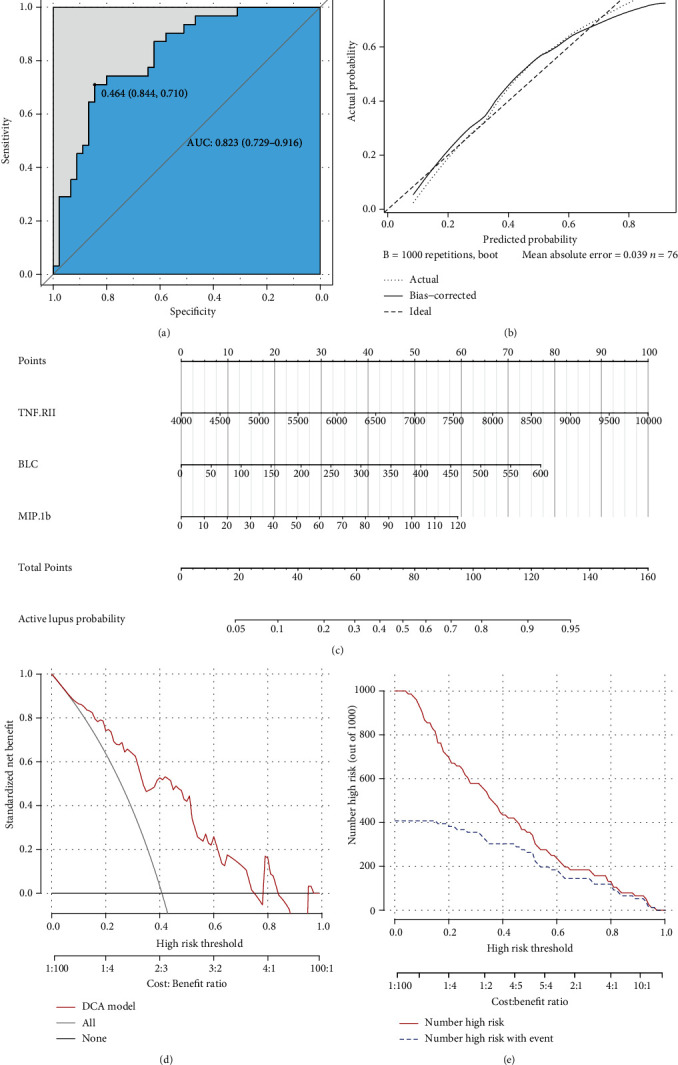
Efficiency and validation of the activity diagnosis model. (a) ROC of activity model in differentiating active SLE from inactive ones. (b) Calibration curve of activity model. (c) Nomogram of activity model with TNF RII, BLC, and MIP-1b. (d) Decision curve for the activity model in predicting active lupus. The red line represented activity model, grey line represented the assumption that all patients suffered active lupus, and black line represented the assumption that all patients had inactive lupus. (e) Clinical impact curve analysis diagram. Red line represented number of patients which were diagnosed with active lupus by our model at different threshold probability, and blue line represented number of patients with active lupus.

ROC analysis was carried out and suggested that this model had a rather good predictive value with an AUC of 0.823 (95% CI 0.729-0.916) and cutoff point of 0.464 (specificity 0.844 and sensitivity 0.710) (Figure 3(a)). The calibration curve showed a mean absolute error of 0.039, which also gave us confidence that this model was qualified and might be helpful in clinical treatment (Figure 3(b)). A nomogram was also presented to make this model convenient to use (Figure 3(c)).

As the assessment method that had been used on the diagnosis model, we also performed decision curve analysis and plotted a clinical impact curve (Figures 3(d) and 3(e)). The DCA model curve suggested that our activity model would benefit us more in a wide range of thresholds. The clinical impact curve showed that we would have more confidence in diagnosing active lupus while we had a relatively higher risk threshold. Both of them implied that the activity model might be promising in clinical decision-making.

### 3.4. Combined Diagnosis Efficiency of Inflammatory Proteins and Conventional Biomarkers in Distinguishing Active Lupus

**Figure 4:**
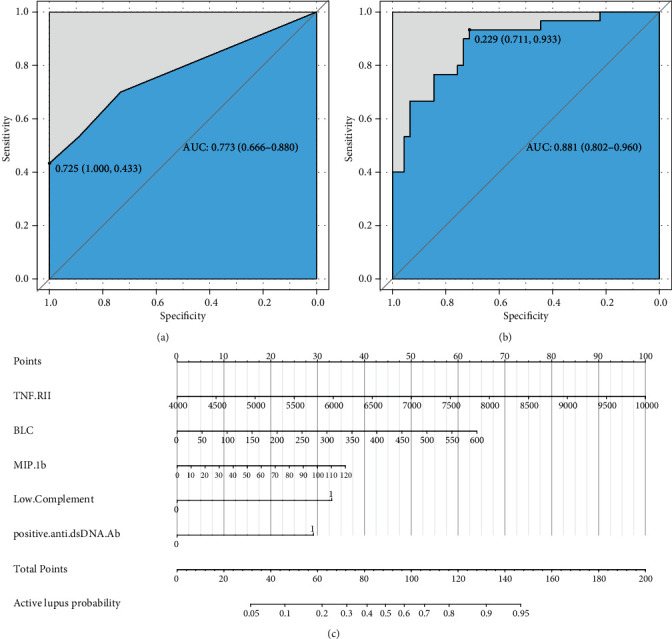
Additional value of our biomarkers based on conventional biomarkers. (a) Conventional biomarkers in predicting active lupus. (b) ROC of combined factors in predicting active lupus. (c) Nomogram of combined activity diagnosis model.

Complement 3 (C3) and 4 (C4) levels and anti-double-stranded DNA antibody (anti-dsDNA Ab) were considered efficient and conventional biomarkers of active lupus and were components of SLEDAI. We performed ROC curve analysis based on low level complements (low levels of C3 and/or C4) combined with positive anti-dsDNA Ab to assess the efficiency of conventional biomarkers in predicting active lupus (SLEDAI ≥ 5) (Figure 4(a)). It showed a moderate AUC value (0.773, 95% CI 0.666-0.880). Then, we combined factors of the activity diagnosis model with this conventional model and formed a novel model of recognizing active lupus, which contained factors of TNF RII, BLC, MIP-1b, low level complements, and positive anti-dsDNA Ab. The ROC curve was also plotted, and it showed an elevated AUC value of 0.881 with a 95% CI of 0.802-0.960 (cutoff point value 0.229, specificity 0.711, and sensitivity 0.933) (Figure 4(b)). For better acceptability, we made a nomogram that could be used in the clinic easily (Figure 4(c)). It contained these five factors that we had mentioned, and we hoped that it could add more benefit and convenience for our clinical decision and treatment.

## 4. Discussion

Currently, an increasing number of SLE patients are being diagnosed with advanced medical inspection technology. However, the disease remission rate is still not where we want to see it, although the overall rate of SLE patients who have achieved and maintained remission status has improved considerably over recent decades, and further evidence is required to better characterize the pathogenesis and features of SLE, especially in patients in the Asia Pacific region [16, 17]. Discovery of new biomarkers of SLE has always been positive, as it can provide better comprehension of SLE pathogenesis and could also be a potential treatment target, for example, CD163, which has been a hot topic lately [18, 19]. Nevertheless, this seems to provide limited value to our clinical decisions and treatment, although it would indeed add more benefit in choosing study topics and developing novel treatment targets. Thus, we aimed to find biomarkers that can benefit SLE diagnosis and predict disease activity precisely in the clinic.

Differential diagnosis and prediction combined with multifeature models have been developed rapidly in recent years, and this method has also been applied in diagnosing SLE. Diagnostic models based on clinical data and manifestations have been developed for better accuracy in diagnosing SLE, including indices such as malar rash, serositis, neurologic disorder, and low C3 and C4 levels [20]. Additionally, it has been reported that RNA could be applied in establishing a risk prediction model of SLE, and they showed high efficiency [21–23]. Models for predicting lupus disease activity that have been developed with combined clinical features have also appealed to us, as well as diagnosing SLE complications and predicting treatment response [24–27]. Thus, we were inspired to establish an SLE diagnosis model from a different perspective. Inflammatory proteins such as cytokines and chemokines have always been considered to play crucial roles in SLE pathogenesis, and multiple protein markers are widely studied worldwide [28]. To discover and validate as many inflammatory proteins as we could efficiently, we performed protein array quantitation and developed an SLE diagnosis model and disease activity diagnosis model. To our excitement, our models showed great differential diagnostic efficiency with AUCs of 0.914 and 0.823, respectively. Additionally, the protein markers we selected could add more benefit to conventional biomarkers, such as C3, C4, and anti-dsDNA Ab, in predicting active lupus. To the best of our knowledge, this is the first study to attempt to establish a diagnosis model and disease activity diagnosis model based on such a wide range of inflammatory proteins. Furthermore, our models might be a bridge between the laboratory and the clinic and could make our experimental findings benefit clinical decisions conveniently. Thus, we could obtain more feedback and further improve our decision models and eventually add more benefit to clinical treatment.

Efficient plasma biomarkers have been explored for a long time because of the multiple varieties of proteins contained and convenience to be obtained, and a few available reports have discovered some promising proteins. The tumor necrosis factor (TNF) superfamily contains proteins with proinflammatory and anti-inflammatory activity and has been studied in many diseases, especially in some rheumatic diseases, such as SLE [29]. They have been proven to play an important role in disease pathogenesis by enhancing the inflammatory activity of immune cells as well as tissue cells themselves and creating a sustained inflammatory microenvironment, which would cause tissue and organ injuries. In addition, some of the TNF superfamily proteins promote cell death and limit inflammation. Novel biologic drugs, such as BAFF antagonists, that could help in the treatment of SLE patients also imply that proteins of the TNF superfamily might be promising biomarkers. Colony stimulating factors (CSF), represented by granulocyte CSF, macrophage CSF, granulocyte-macrophage CSF, and a few other cytokines, have been proven to be upregulated when infections and autoimmune diseases occur. They can enhance the activity of granulocytes, macrophages, and both granulocytes and macrophages and promote their cell proliferation, as well as monocyte chemoattractant proteins (MCP) [30]. It is well known that SLE damage is mainly caused by an immune complex-mediated autoimmune response, and patients with SLE usually have impaired macrophage function, which could result in a reduction in macrophage phagocytotic ability, and then, an increasing number of deposited immune complexes are left, increasing injuries [31]. Additionally, CSF could stimulate the activation and migration of macrophages and granulocytes to inflammation sites and sustain their survival and renewal, and an imbalance in CSF production would result in harmful effects. However, some reports have suggested protective roles of CSF in SLE [32, 33]. Thus, we were interested in what roles they played in patients with SLE. We could never ignore interleukins (IL) when talking about abnormal cytokines in SLE patients. ILs have been discussed in many reports and used as a treatment target owing to the variety of types and broad biological significance and signaling pathways. Similar to TNFs, ILs do not always promote immune responses, but they can regulate immune cell activity; thus, IL-2 can be used for SLE treatment, as well as IL-12 and IL-17, which are promising therapeutic targets, as well as potential biomarkers of disease activity [34–36]. Moreover, gene polymorphisms of a few ILs have also been proven to influence the predisposition of SLE [37]. All of these inflammatory proteins attracted our interest.

Therefore, we chose the inflammation array Cat# QAH-INF-G3-4 to quantify 40 proteins in our plasma samples, including TNF RI, TNF RII, BLC, MIP-1b, eotaxin, eotaxin-2, MIG, MCSF, G-CSF, GM-CSF, MCP-1, MIP-1a, MIP-1d, PDGF-BB, TIMP-1, TIMP-2, I-309, TNFa, TNFb, RANTES, IFNg, ICAM-1, IL-1a, IL-1b, IL-1RA, IL-2, IL-4, IL-5, IL-6, IL-6R, IL-7, IL-8, IL-10, IL-11, IL-12p40, IL-12p70, IL-13, IL-15, IL-16, and IL-17, and samples from both SLE patients and healthy controls were quantified.

Proteins that we quantified were considered statistically significant not only based on their low *p* value but also with FD > 1.2 (upregulated) or FD < 0.83 (downregulated). Additionally, to reduce statistical error in distinguishing differentially expressed proteins, we calibrated our *p* value using the Benjamini and Hochberg (BH) method and generated an adjusted *p* value. Thus, we could recognize proteins that satisfied our demands visually in the volcano plot underlying both adjusted *p* value and FD. Following univariable and multivariable logistic regression analysis, TNF RII and MIP-1b were enrolled in the diagnosis model, and TNF RII, BLC, and MIP-1b were enrolled in the active lupus diagnosis model.

Tumor necrosis factor receptor type II (TNF RII) belongs to the tumor necrosis factor receptor superfamily and is bound to the cell membrane, and it can enhance inflammatory injuries induced by infections and autoimmune diseases [38]. According to available studies, the serum-soluble TNF RII level was higher in patients with SLE than in healthy volunteers, not only at the time of diagnosis but also at the time of posttreatment, and it showed a significant correlation with lupus disease activity [39, 40]. Additionally, the TNF RII level was associated with a decreased estimated glomerular filtration rate in SLE patients with renal involvement. This evidence implied that TNF RII might take part in lupus pathogenesis and organ injuries. B lymphocyte chemoattractant (BLC), also known as CXC motif ligand 13 (CXCL13), and its corresponding receptor CXCR5 have been reported to be part of SLE pathogenesis and the levels of BLC were higher in SLE patients than in healthy controls [41]. Additionally, lupus patients with higher disease activity scores showed higher circulating BCL levels [42]. Regarding macrophage inflammatory protein 1 beta (MIP-1b), some debate remains. MIP-1b, also known as chemokine (CC motif) ligand 4 (CCL4), is a powerful chemokine and can enhance immune system disorders by recruiting regulatory T cells and macrophages, which eventually results in organ lesions [43]. In some studies, MIP-1b might be a promising biomarker of SLE underlying its ability to predict active lupus, and circulating MIP-1b levels were higher in patients with SLE than in healthy controls [44, 45]. However, quite a few studies showed negative opinions and suggested that there was no MIP-1b difference between patients with SLE and healthy persons [46]. Thus, we concluded that MIP-1b has the potential to be a qualified biomarker of SLE.

Our models were validated to be efficient, and the DCA curve and clinical impact curve showed that the models could add more benefit to our clinical decision and treatment strategies. Clinicians would have more confidence in diagnosing SLE if our diagnosis model could be well used. Moreover, how to monitor lupus activity precisely and conveniently has troubled us for a long time. The full-scale current evaluation rules are complicated and costly, and this urges us to develop a novel method to be used in the clinic. This disease activity diagnosis model not only showed high efficiency but could also provide additional benefit to conventional biomarkers in differentiating active lupus from inactive ones. It would be a promising tool in helping develop an appropriate treatment plan and provide more evidence in judging treatment response and taping drug doses.

Our study developed diagnosis and activity diagnosis models based on forty types of inflammatory proteins. We then validated the model efficiency, and it showed high value in contributing to clinical determinations. We obtained a negative outcome when we further performed subgroup analysis to verify whether the protein levels were significantly different between SLE patients with or without renal involvement. In the subgroup analysis, a few protein levels were significantly higher than those of healthy controls, with an original *p* value less than 0.05, whereas all of the adjusted *p* values were more than 0.05. Additionally, our evaluations had not been performed blindly, which might potentially make our findings slightly less convincing, and we could have done it better if we had carried out our analysis blindly. Last, we have no follow-up data at present, but we will follow up with these patients and obtain more data to determine whether we could develop a model that could predict the disease flare and prognosis of our SLE patients and eventually benefit their lives.

## 5. Conclusions

In this study, we developed a disease diagnosis model and disease activity diagnosis model based on the inflammatory array quantitation data of forty proteins, and both of these models have been verified to be efficient and convenient for application in clinical decisions. We believe that they would have promising performance and benefit clinicians and our patients.

## Figures and Tables

**Table 1:** General clinical characteristics of population.

Variable	SLE (*N* = 76)	Control (*N* = 21)	*p* value
Age (year), median (IQR)	30.0 (24.0-44.8)	25.0 (22.5-27.5)	0.009
Gender, *n* (%)			0.138
Male	12 (15.8%)	7 (33.3%)	
Female	64 (84.2%)	14 (66.7%)	
Ethnic origin	Middle of China	Middle of China	—
Onset age (year), median (IQR)	28.0 (21.3-38.8)	—	
Disease duration (month), median (IQR)	15.5 (2.0-43.9)	—	
SLEDAI		—	
Median, IQR	4 (1-10)		
0-4, median (IQR)	45 (59.2%)		
≥5, median (IQR)	31 (40.8%)		
Positive anti-ANAs, *n* (%)	69 (90.8%)	—	
Positive anti-dsDNA Ab (%)	22 (28.9%)	—	

**Table 2:** Proteins which showed statistical differences between the SLE group and control group.

Protein ID	AveExp. SLE	AveExp. Con	Fold change	log2 (FD)	*p* value	Adjusted *p* (BH)	Regulation
TNF RII	8.75426	8.51666	1.27915	0.35519	4.74E-13	1.897E-11	Up
BLC	3.73913	2.60778	3.44763	1.78560	1.60E-05	0.00016	Up
TNF RI	8.93266	8.62099	1.40409	0.48964	1.20E-05	0.00016	Up
MIP-1b	3.28035	2.52714	2.44976	1.29264	1.10E-05	0.00016	Up
Eotaxin	4.89815	4.30996	1.88399	0.91379	2.50E-05	0.00020	Up
MIG	3.00623	1.56953	15.48548	3.95284	7.07E-04	0.00354	Up
MCSF	0.95948	-1.00187	35.34142	5.14329	1.64E-03	0.00657	Up
IL-8	-0.61980	-1.85654	3.18792	1.67262	5.45E-04	0.00311	Up
MCP-1	4.53743	4.04290	1.84726	0.88538	8.46E-04	0.00376	Up
IL-10	0.01865	-1.10739	2.57508	1.36462	3.47E-04	0.00231	Up

**Table 3:** Logistic regression analysis in differentiating SLE patients from healthy controls.

Proteins	Univariable		Multivariable	
	OR (95% CI)	*p*	OR (95% CI)	*p*
TNF RII	1.002 (1.001-1.003)	<0.001	1.002 (1.001-1.003)	<0.001
BLC	1.025 (1.004-1.047)	0.021		
TNF RI	1.001 (1.000-1.001)	<0.001		
MIP-1b	1.126 (1.046-1.212)	0.002	1.105 (1.007-1.212)	0.035
Eotaxin	1.021 (1.009-1.033)	<0.001		
MIG	1.106 (1.016-1.204)	0.020		
MCSF	3.443 (0.965-12.282)	0.057		
IL-8	4.901 (1.144-21.000)	0.032		
MCP-1	1.021 (1.007-1.035)	0.004		
IL-10	1.027 (0.902-1.168)	0.692		

**Table 4:** Logistic regression analysis of differentiating active SLE from inactive SLE.

Variable	Univariable		Multivariable	
	OR (95% CI)	*p*	OR (95% CI)	*p*
TNF RII	1.001 (1.000-1.001)	0.002	1.001 (1.000-1.001)	0.006
BLC	1.007 (1.001-1.013)	0.032	1.007 (1.001-1.012)	0.020
TNF RI	1.000 (1.000-1.000)	0.049		
MIP-1b	1.031 (1.008-1.053)	0.007	1.026 (1.002-1.050)	0.035
Eotaxin	1.001 (0.995-1.007)	0.749		
MIG	1.000 (0.997-1.004)	0.792		
MCSF	1.030 (0.974-1.090)	0.299		
IL-8	1.616 (0.968-2.700)	0.067		
MCP-1	1.006 (1.000-1.013)	0.060		
IL-10	0.990 (0.954-1.027)	0.593		

## Data Availability

The data that support the findings of this study are available on request from the first author and corresponding author underlying reasonable request.
